# Increased body movement equals better performance? Not always! Musical style determines motion degree perceived as optimal in music performance

**DOI:** 10.1007/s00426-024-01928-x

**Published:** 2024-02-08

**Authors:** Nádia Moura, Pedro Fonseca, João Paulo Vilas-Boas, Sofia Serra

**Affiliations:** 1 Research Centre in Science and Technology of the Arts (CITAR), School of Arts, Universidade Católica Portuguesa, Porto, Portugal; 2 Porto Biomechanics Laboratory (LABIOMEP), Faculty of Sport, University of Porto, Porto, Portugal; 3 Centre of Research, Education, Innovation and Intervention in Sport (CIFI2D), Faculty of Sport, University of Porto, Porto, Portugal; 4 Instituto de Etnomusicologia–Centro de Estudos em Música e Dança (INET-MD), Departamento de Comunicação e Arte, Universidade de Aveiro, Aveiro, Portugal

## Abstract

Musicians’ body behaviour has a preponderant role in audience perception. We investigated how performers’ motion is perceived depending on the musical style and musical expertise. To further explore the effect of visual input, stimuli were presented in audio-only, audio–visual and visual-only conditions. We used motion and audio recordings of expert saxophone players playing two contrasting excerpts (positively and negatively valenced). For each excerpt, stimuli represented five motion degrees with increasing quantity of motion (QoM) and distinct predominant gestures. In the experiment (online and in-person), 384 participants rated performance recordings for expressiveness, professionalism and overall quality. Results revealed that, for the positively valenced excerpt, ratings increased as a function of QoM, whilst for the negatively valenced, the recording with predominant flap motion was favoured. Musicianship did not have a significant effect in motion perception. Concerning multisensory integration, both musicians and non-musicians presented visual dominance in the positively valenced excerpt, whereas in the negatively valenced, musicians shifted to auditory dominance. Our findings demonstrate that musical style not only determines the way observers perceive musicians’ movement as adequate, but also that it can promote changes in multisensory integration.

**Supplementary Information:**

The online version contains supplementary material available at 10.1007/s00426-024-01928-x.

## Introduction

In music performance, like other forms of social interaction, nonverbal behaviour is responsible for communicating considerable amounts of information (Cordaro et al., 2019; Watson & Gelder, 2019; Witkower et al., 2021). Seeing the performer is a central motivation factor for preferring live performances to recordings, which are often flawless and high quality (Bergeron & Lopes, 2009; Cook, 2008; Platz & Kopiez, 2012). Consequently, meaningful interactions rely on the successful encoding and decoding of expression, respectively, by the musicians and the audience (Juchniewicz, 2008; Leman, 2016). Musical expression, in turn, encompasses not only auditory elements such as rhythmic variations (Huberth et al., 2020; Repp, 1992) or timbre (Li & Timmers, 2020), but also non-auditory cues related to the facial and bodily actions of the performers (Davidson, 2012; Laeng et al., 2021; Nápoles et al., 2022; Weiss et al., 2018). Although body movement is not the ultimate goal in music performance, as it is in dance, there has been a growing interest in this dimension, considering that movements significantly affect observers’ opinion (Bugaj et al., 2019; Nápoles et al., 2022; Silveira, 2014; Trevor & Huron, 2018; Wapnick et al., 2004) and auditory perception (Juchniewicz, 2008; Nápoles et al., 2022; Schutz & Lipscomb, 2007; Wapnick et al., 2004). The relevance of studying how nonverbal behaviour influences music performance perception is multifaceted, ranging from the understanding of processes underlying social connectedness to the multisensory integration involved in associating auditory and visual stimuli. Ultimately, it also contributes to the development of pedagogical instructions on body awareness for music practitioners.

### The role of visual cues

Research has demonstrated that visual cues alone are effective in conveying information about music performances, such as who are the most expressive musicians (Davidson, 1993) or competition winners (Tsay, 2013, 2014). Nevertheless, when coupled with sound, visuals still play a decisive role in manipulating or enhancing auditory cues (Behne & Wöllner, 2011; Broughton & Stevens, 2009; Coutinho & Scherer, 2017; Id et al., 2019; Lange et al., 2022; Tsay, 2013). For example, when presented with varied performance videos with the same audio, even musically trained participants perceived sonic differences (Behne, 1990; Behne & Wöllner, 2011). In fact, visual dominance has been reported in the evaluation of sound-related aspects such as rhythmic accuracy or tone quality (Pope, 2019; Wapnick et al., 2004), intensity and emotion (Lange et al., 2022), expressiveness (Broughton & Stevens, 2009), and in the emotional reaction of the audience (Coutinho & Scherer, 2017). In line with the previous studies, Li and Colleagues (2021) found that visual information determined pianists’ efficiency in communicating timbral intentions of tension and relaxation. However, the communication of other timbral intentions was revealed to be heavily dependent on the performer (Li et al., 2021).

The congruency of audio and visual stimuli can, therefore, assume an enhancing perceptual effect. In a study by Schutz and Lipscomb (2007), participants distinguished note durations through visual integration, when percussive sounds were accompanied by gestures, and not through acoustic information alone. Comparably, Thompson and Colleagues (2005) concluded that musical dissonances were better conveyed when presented alongside facial expressions, and hardly differentiated from the neutral stimuli in the audio-only presentations. In the field of emotion, physiological responses were more intense when happy and sad music was congruent with facial pictures (Id et al., 2019). Thus, understanding how observers perceive musicians’ body behaviour as adequate to the sonic output enables performers to improve audience engagement and musical comprehension.

When it comes to emotionality, however, sound seems to be prevalent. Auditory dominance has been reported in perceiving affective nuances, such as majestic, passionate or humorous (Shoda & Adachi, 2016; Vines et al., 2011). Van Zijl and Luck (2013) found a small potentiator effect in identifying sadness in sensory modes including auditory information, compared to video-only. Equally, sound-including conditions enhanced participants’ accuracy in detecting the musicians’ expertise level (Rodger et al., 2012). Furthermore, other studies, although reporting main findings of visual dominance, also found mixed interactions between visual and auditory cues, suggesting that the experience of emotions (Coutinho & Scherer, 2017) and expressivity (Vuoskoski et al., 2014) in musical settings is a complex phenomenon that requires beyond systematic investigation. The question emerges: is our perception mostly influenced by what we see, or are there contexts in which music alone can have a stronger impact?

### Influence of body behaviour

Body behaviour is the major component of the visual dimension in music performance. Studies focussed on the role of visuo-kinematic cues in audience perception have addressed two complementary views: full-body motion, tackling musicians’ movement as a general concept involving the body as a whole (Bugaj et al., 2019; Burger & Wöllner, 2023; Davidson, 1993; Moura et al., 2023; Nápoles et al., 2022; Nusseck & Wanderley, 2009; Trevor & Huron, 2018; Van Zijl & Luck, 2013), and motion types, providing a detailed view on specific gestures or body locations (Davidson, 1994; Nusseck & Wanderley, 2009; Weiss et al., 2018).

In the full-body domain, the common finding between studies is that, across several assessment variables, participants give better ratings to performative conditions with increased amounts of movement (Broughton & Stevens, 2009; Bugaj et al., 2019; Burger & Wöllner, 2023; Davidson, 1993; Grady & Gilliam, 2020; Juchniewicz, 2008; Moura et al., 2023; Nápoles et al., 2022; Nusseck & Wanderley, 2009; Silveira, 2014; Van Zijl & Luck, 2013). Accordingly, Küssner and Colleagues (2020) demonstrated that visual attention is directed towards the musicians moving more expressively, even if they are not playing the most relevant musical part. In the study by Trevor and Huron (2018), when asked to adjust the amplitude of motion to create the best animated performances, participants augmented musicians’ motion. Moreover, musicians reported concentrating more on quantity than regularity or velocity of motion to discriminate between gradual expressivity conditions (Massie-Laberge et al., 2016). Regarding emotion recognition in music performance, Dahl and Friberg (2007) found that perceived emotions (i.e. happy, sad) associated with variations in motion properties, including speed, regularity, fluency and amount of movement. Research in full-body dance movements has also validated that kinematic descriptors are key features in basic emotion recognition (Burger & Toiviainen, 2020; Burger et al., 2013; Camurri et al., 2003; Castellano et al., 2007).

Second, motion type studies seek to understand which body locations or gestures are relevant for communication. Such is the case of the head, which has been shown to be particularly effective in communicating expressive (Davidson, 1994; Massie-Laberge et al., 2016) and emotional intentions (Dahl & Friberg, 2007) when compared to other body parts. Whilst the previous studies adopted the paradigm of presenting separate body parts of musicians to observers for assessment, others present musicians with contrasting motion behaviours. For instance, Weiss and Colleagues (2018) included four motion types of clarinettists in their study, in which participants rated five performative aspects (i.e. expressiveness, professionalism). The arm and knee motion types were the highest rated, followed by the type with no prominent motion and the low motion type, which scored the lowest. Contrarily, in Nusseck and Wanderley (2009), nearly no differences were observed in participants’ ratings of four motion profiles (regular, arm-only, torso-only, mismatched motion). Literature covering the perceptual impact of other motion types was not found, even though some are well known to be recurrent amongst musicians, like bell gestures (Teixeira et al., 2014; Wanderley et al., 2005) or swaying (Chang et al., 2019; Davidson, 2012; Demos et al., 2017).

In sum, although it has been argued that the ability to grasp musical attitudes is mostly dependent on the overall motion patterns of musicians rather than isolated gestures (Castellano et al., 2007; Nusseck & Wanderley, 2009), due to the small number of studies focussed on motion types, further research is required to better understand this phenomenon. Furthermore, considering that full-body motion studies commonly use two to four movement conditions, research including expanded conditions of varying motion patterns is necessary (Silveira, 2014), which led to the development of the current study.

### Evidence on mediators of perception: musical expertise and musical style

Musicians develop enhanced multisensory processing, related to the simultaneous visual, auditory and motor processes involved in long-term instrument playing (Ihalainen et al., 2023; Paraskevopoulos et al., 2015; Zatorre et al., 2007). In addition, in listening aesthetic judgements, musicians rely on a larger number of criteria than non-musicians (Juslin, 2013; Juslin et al., 2021). If so, similarly to what occurs in dance (Vinken & Heinen, 2022), shouldn’t music performance perception be mediated by musical expertise? Evidence is controversial. Whilst some studies suggest that audiences without musical instruction rely more on visual than aural cues in evaluation (Davidson, 1995; Davidson & Correia, 2002; Huang & Krumhansl, 2011), others demonstrate that trained musicians make similar (Tsay, 2013, 2014) and stronger use of vision (Lange et al., 2022). Yet, although Tsay (2013, 2014) confirmed that both experts and laypersons were able to identify competition winners based on video alone, a recent direct replication of the 2014 study proved that novices were unable to perform the task at a greater level than chance, regardless of the sensory mode (Wilbiks & Yi, 2022).

Studies have shown that musicians are better able to detect mismatches between movement and sound (Weiss et al., 2018) and tend to give higher expressivity and interest ratings than non-musicians (Broughton & Stevens, 2009), but also that no significant differences emerge between both in emotion (Shoda & Adachi, 2016; Vines et al., 2011) and expertise (Rodger et al., 2012) assessments. Within highly trained musicians, perceptual rating differences were not found between music undergraduate vs. graduate students (Juchniewicz, 2008) nor between different instrumentalists (Nusseck & Wanderley, 2009). However, one study revealed that expert singers preferred average movement levels, whilst the less experienced preferred increased movement (Grady & Gilliam, 2020). Although the perceptual constructs amongst musicians seem concordant, further research is needed to better understand if and how they differ from non-musicians.

Another potential mediator factor in performance perception, although often neglected, is musical style. Aspects like tonality (López & Anta, 2023) or rhythm (Senn et al., 2017; Varlet et al., 2020) have a significant influence on listeners. Nonetheless, by adopting designs including one musical piece (Moura et al., 2023; Nápoles et al., 2022; Nusseck & Wanderley, 2009; Weiss et al., 2018) or grouping a large number of excerpts together in the same analysis (Küssner et al., 2020; Lange et al., 2022), studies are unable to address the influence of the repertoire. Conversely, an effect of musical style has been reported in studies accounting for this variable (Burger & Wöllner, 2023; Huang & Krumhansl, 2011; Shoda & Adachi, 2016; Wapnick et al., 2004). Huang and Krumhansl (2011) found that the ratings of baroque and romantic music increased as a function of stage behaviour, whereas minimal and natural behaviour was preferred for modern music. Complementarily, in the study by Trevor and Huron (2018), augmented movement was preferred for fast technical passages and only slightly above normal movement for slow, lyrical passages. According to Wapnick and Colleagues (2004), romantic and modern excerpts received higher ratings from pianists (vs. non-pianists) than classical excerpts. Also, compared to music undergraduates, graduate/faculty participants judged fast excerpts more severely than slow ones. When compared to slow music, energetic music has also been perceived as more expressive (Burger & Wöllner, 2023) and allowed for better accuracy in identifying expressive conditions (Shoda & Adachi, 2016). Hence, these findings not only reinforce that individual music preferences interfere with participants’ evaluation but also that the body behaviours perceived as optimal vary according to the repertoire.

## Present study

The main aim of our study was to investigate how musicians’ body behaviour contributes to the perception of the overall quality of music performance. Specifically, we wanted to understand how the preferred motion degrees differed depending on the musical repertoire and musical expertise, and whether observers relied more on the quantity or quality of motion for their evaluation. Previous research demonstrated that increased amounts of movement associate with increased performance ratings (e.g. Bugaj et al., 2019; Davidson, 1993; Nusseck & Wanderley, 2009, see Introduction for more details). However, given that most studies use two (Moura et al., 2023; Nápoles et al., 2022) or three motion styles (Silveira, 2014; Van Zijl & Luck, 2013), it is natural that participants incline towards upvoting the condition with larger movement and vice versa. To better understand this phenomenon, we expanded the number of motion degrees included in the design (five for each of the two musical excerpts). These were thoroughly selected to represent gradual levels of global quantity of motion (QoM), calculated from the motion data. Hence, the first motion degree, D1, corresponded to minimal movement, and the last, D5, to exaggerated movement. To assess the influence of motion quality, in the in-between motion degrees (D2…D4), we included performances presenting predominant gesture types (i.e. flap, head nod).

We hypothesised that, if observers relied more on the quantity of motion, ratings would increase as a function of the QoM values (Hypothesis 1a). Contrarily, if observers relied more on the quality of motion, the ratings of one or more in-between motion degrees presenting a prominent gesture type (D2…D4) would surpass the exaggerated degree (D5) (H1b).

Regarding musical style, we used two contrasting pieces representing positive and negative valence. In Western classical music, fast tempo and major modes are associated with positive valence (i.e. happiness, joy), and slow tempo and minor modes with negative valence (i.e. sad) (Husain et al., 2002; Schellenberg et al., 2008; Thompson et al., 2001; Webster & Weir, 2005). Therefore, following previous findings (Trevor & Huron, 2018), we hypothesised that the “optimal motion degree” would differ according to the musical style: increased motion would be preferred for the energetic, joyous excerpt, and average to low motion would be preferred for the slow-paced melancholic excerpt (H2). We further considered that ratings would differ between musician and non-musician groups, with the latter associating exaggerated motion conditions with better performance (H3).

Second, we wanted to revisit the role of the visual component in music performance perception to validate whether body behaviour is, in fact, fundamental for audience engagement. For that, based on previous research (i.e. Coutinho & Scherer, 2017; Davidson, 1993; Lange et al., 2022), we presented the set of ten stimuli in audio-only (A), audio–visual (AV) and visual-only (V) conditions. Here, we hypothesised that patterns of visual dominance would emerge in both excerpts (H4). Regarding expertise, considering the existing contradictory findings, we predicted that either non-musicians would make more use of visual cues (Davidson, 1995; Davidson & Correia, 2002; Huang & Krumhansl, 2011) (H5a), the inverse (Lange et al., 2022) (H5b), or not to find significant differences between groups (Tsay, 2013, 2014) (H5c). In addition, it would be expected that the AV condition would collect the highest ratings due to its richer multisensorial nature, considering that presentations of congruent audio–visual stimuli enhance perception when compared to unimodal conditions (Id et al., 2019; Thompson et al., 2005).

## Method

### Participants

Participants were recruited via convenience and snowball sampling by email, social media and online survey platforms. All participants reported having ≥ 18 years old, normal hearing and normal or corrected-to-normal vision. In the initial information sheet, a disclaimer was included to indicate that both musicians and non-musicians were eligible. Although we collected data from 201 musicians and 203 non-musicians, some exclusions were performed to improve the reliability of our sample: 9 participants were excluded due to missing data; 5 participants due to extensive duration of participation and 6 non-musician participants who reported less than 6 years of formal music training (we preferred to keep a non-musician group who only had general music classes or none).

The final sample included 384 participants. They were divided into 2 groups (192 participants each) according to their musical background, following the “musical expertise criterion” of having a minimum of 6 years of music training to be considered a musician (Zhang et al., 2020). The musically trained group (113 female, 76 male, 3 other, age *M* = 29.33, SD = 9.49, range 18–62 years old) reported an average of 13.28 years of music training (SD = 7.56, range 6–52 years). This group included 96 professional musicians (50%), 83 participants who received tuition in a music school, academy, or conservatoire (43.2%), and 13 who had private instrument classes (6.8%). In the untrained group (117 female, 70 male, 5 other, age *M* = 27.65, SD = 8.81, range 18–61 years old), 123 participants had general music lessons in regular school (64.1%), 53 never had music lessons or practise (27.6%), and 16 never had music lessons but considered themselves self-taught musicians (8.3%).

The study was approved by the Comissão de Ética para a Saúde of the Universidade Católica Portuguesa under protocol number 196/2022. Participants provided their informed consent at the beginning of the experiment.

### Stimuli: selection and preparation of trials

The stimuli were retrieved from a pre-existing database, including motion and audio recordings of 20 expert saxophone players (for details regarding data collection, see Moura et al., 2022). First, authors NM and SS consensually selected two highly contrasting musical excerpts from the database based on the criteria of fitting the temporal and modal constructs associated with positive and negative-valenced music validated by previous research (Husain et al., 2002; Schellenberg et al., 2008; Thompson et al., 2001; Webster & Weir, 2005). The selected excerpts were bars 14–20 of *Rhapsodie* by Debussy (1998) and bars 1–26 of the 3rd movement (“with gaiety”) of *Sonata Op. 19* by Creston (1945) (Fig. 1 of SI). Representative of negative valence, the Debussy excerpt illustrates a melancholic, mysterious character in an *ad. Libitum* slow tempo. Its melodic gestures follow an heptatonic organisation characteristic of Debussy’s works, departing from the traditional diatonic system and, therefore, likely to sound unfamiliar to the ear of the common listener (Laneve et al., 2023). On the other hand, the Creston excerpt presents a rhythmical, energetic character, is fast-paced and is composed in a major tonality, hence following the features associated with positive-valenced music.

Initially, for each excerpt, we had available recordings of 20 performers. However, we wanted to reach the planned five recordings per excerpt, representing increasing motion degrees. For that, we calculated the quantity of motion (QoM) based on the norm of 3D velocities, following the routines reported in other music and motion studies (Bishop et al., 2021; Gonzalez-Sanchez et al., 2018). A cautionary note should be made, as the concept of quantity of motion used in music and movement studies differs from the one used in biomechanics, also known as linear momentum, obtained by multiplying the velocity vectors by the segment masses (Robertson et al., 2014). Here, we adopted the first to follow common practises within the field. We used the Matlab Mocap toolbox (Burger & Toiviainen, 2013) to extract position data, compute velocity (first derivative) and consequent norm of velocity from the three components of the vector. The resulting values were then averaged, resulting in the QoM for the following body locations: head, torso, sax bell, (left and right) elbow, knee and foot. To produce a global QoM per performer, the values of the previous body locations were averaged together. This allowed us to have a movement descriptor of the full body (Global QoM) and of multiple body parts, further enabling us to order the trials from minimum to maximum QoM.

Second, we selected the trials with the lowest (D1) and highest (D5) global QoM for each musical excerpt. For the other in-between trials (D2…D4), we visually inspected the recordings to find performers who consistently executed specific motion types and examined the QoM values associated with their corresponding body parts. Nevertheless, we realised that the local QoM not always reflected the prevalent gesture of the trial. For example, a performer who head nodded repeatedly whilst moving minimally the rest of the body presented lower head QoM than a performer who swayed sideways, thus moving the head along with the rest of the body. Yet, to the naked eye, the head movement was characteristic of the first performer and not of the second. In this sense, for D2…D4, we decided to rely on the systematic observation of the remaining trials and selected the performers who illustrated prominent gesture types. Authors NM and SS analysed the trials individually and met to reach a consensus regarding the trials that were more representative of particular gestures. A description of the final ten trials selected is presented in Table 1, with information regarding quantity (QoM) and quality (gesture description) of motion. In addition, we added the local QoM values to the Supplementary Information (Table 1 of SI), as well as example videos of the audiovisual stimuli with the highest and lowest ratings per excerpt (SI Video 1…4).

**Table 1:** Characterisation of the motion degrees used in this study

Musical excerpt	Motion degree	Name	Global QoM (mm/s)	Qualitative motion type description
Debussy	D1	Minimal	40.91 ± 23.93	Minimal motion with no prominent gestures
	D2	AP Sway	69.88 ± 40.41	Prominent anteroposterior sway with one foot placed in front of the other and constant weight transfer back and forth
	D3	ML Sway	87.92 ± 53.47	Prominent mediolateral sway with both feet aligned at the distance of the shoulders and constant weight transfer from one to the other
	D4	Flap	97.36 ± 65.62	Prominent left arm flap (adduction and abduction of the left arm with flexed elbow)
	D5	Exaggerated	101.62 ± 59.73	Exaggerated motion involving multiple gestures combined
Creston	D1	Minimal	24.86 ± 14.27	Minimal motion with no prominent gestures
	D2	Head Nod	80.39 ± 39.74	Low overall motion with prominent head nods
	D3	Trunk + Knee Flexion	133.63 ± 75.83	Prominent flexions of the trunk accompanied by the knees
	D4	Flap	148.44 ± 95.71	Prominent left arm flap (adduction and abduction of the left arm with flexed elbow)
	D5	Exaggerated	204.04 ± 120.55	Exaggerated motion involving multiple gestures combined

QoM (quantity of motion) is presented as mean ± standard deviation

The final stimuli set comprised recordings of ten saxophone players (mean age: 25.6 ± 3.8 years old; average years of formal saxophone instruction: 16.1 ± 4.2). Motion files were processed in Visual3D v6 to generate a skeleton avatar for standardisation and control of external confounders, such as physical appearance or dress style of the performers (Griffiths, 2008; Urbaniak & Mitchell, 2022). We selected the skeleton, as more anthropomorphic visual displays perform better at conveying expressiveness than simplified displays like stick-figures (Moura et al., 2023). Then we used Open Broadcaster Software to video record motion files and DaVinci Resolve 17 to synchronise the videos with the original audio tracks. To normalise between-files volume, audio tracks were equalised. The duration of the stimuli ranged between 21 and 28 s in the Debussy and between 19 and 22 s in the Creston. This resulted in a final set of 30 trials for evaluation: 10 audio (A) tracks, 10 audio–video (AV) clips, and 10 video-only (V) clips.

### Procedure

The experiment was conducted via Qualtrics platform in two modes: online (66.1% of the participations of the musician group, 76% of non-musician group) and in-person (respectively, 33.9% and 24% of the participations). Although it could be conducted fully online, we considered that having part of the sample participate under controlled conditions would increase the reliability of the study. In the online version, we adopted a design compatible with portable devices to reach a higher number of valid responses. Research shows that respondents provide conscientious answers on smartphones as likely as on computers when the question designs fit the dimensions of reduced screens (Antoun et al., 2017). In the in-person sessions, participants responded individually using a laptop and headphones. The data collection took place between July and October 2022.

The survey began with an information sheet followed by the informed consent. A group of sociodemographic questions was then presented, followed by the evaluation of the stimuli. All participants evaluated all 30 stimuli (3 sensory modes * 2 musical excerpts * 5 motion degrees). Excerpts were presented in three randomised blocks of ten trials, one per sensory mode (A, AV, V). The conditions were also randomised within blocks and presented on individual pages. To ensure participants listened to/watched the full excerpts, we introduced a 30-s timer starting from the first click; follow-up arrows only appeared after this count. In the A block, a player bar appeared, and in the AV and V blocks, videos were presented centrally in square format (the video itself had a black background and the page a white background).

Excerpts were evaluated through three horizontal sliders presented below the clips, corresponding to the assessment variables of expressiveness, professionalism and overall quality. These aspects were retrieved from previous literature (e.g. Davidson, 1993; Nusseck & Wanderley, 2009; Platz & Kopiez, 2012; Weiss et al., 2018). Each slider presented seven levels, analogous to a seven-point Likert scale, commonly used in music perception studies (e.g. Bugaj et al., 2019; Davidson, 1993; Lange et al., 2022; Wapnick et al., 2004). Labels of “1—Not at all” and “7—Extremely” were presented, correspondingly, on the left and right ends of the sliders. The initial handle was placed at the middle point of the bar to avoid bias caused by initially positioning handles at extreme points (Bosch et al., 2019; Funke et al., 2011; Maineri et al., 2021). When the handle was moved, numeric feedback was provided. Responses required at least one click in the slider for recording. Debriefing and contact information was provided on the last page of the survey.

### Data analysis

An a priori power analysis was run using G*Power 3.1 (Faul et al., 2007) to determine the minimum sample size required for this study. This estimate considered the planned use of two multivariate analyses of variance (MANOVAs) with within–between interaction, an alpha level of 0.05, and a large effect size of 0.8 (Cohen, 1988). For the test with the highest number of cells in the design, we needed at least 79 participants to achieve a power of 95%.

To arrive at a composite score for performance evaluation, we preliminary tested inter-correlations between the rating scores of expressiveness, professionalism, and overall quality for each cell of the design. We found strong Pearson paired correlations between the three evaluation aspects (*r* > 0.5, after Cohen, 1988) in all ten stimuli, in the three sensory conditions, for the musician and non-musician groups (all correlation coefficients are detailed in Table 2 of SI). Therefore, we decided to average the three scales into a composite score representing the overall performance evaluation (OPE), following previous studies (Juchniewicz, 2008; Lange et al., 2022).

To test for Hypotheses 1…3 (see present study), we performed a 2 × 2 × 5 multivariate analysis of variance (MANOVA). The two groups of musical expertise (M: musician, NM: non-musician) were entered as the between-subjects variable, the two musical excerpts (C: Creston, D: Debussy) and the five degrees of body behaviour (D1…D5) as the within-subjects variables. The dependent variables were the participants’ overall performance evaluation (OPE) scores for the AV condition (ten stimuli assessed). To test for H4 and H5, we performed a 2 × 2 × 5 × 3 MANOVA, adding the sensory condition (A, AV, V) as within-subject variable, and using as dependent variables the corresponding OPE scores (30 stimuli assessed).

The statistical analyses were performed using the IBM SPSS 28 package. The alpha level for all tests was set at α = 0.05. When the Box’s M result was statistically significant, we reported Pillai’s Trace, as recommended due to its robustness against this violation (Olson, 1976; Tabachnick & Fidell, 2014). Significant main effects and interactions were followed-up through post hoc pairwise comparisons using the Holm–Bonferroni correction. The observed power for each effect (Power = 1 − β err prob) is reported under the abbreviation OP.

## Results

### Effects of body behaviour

The three MANOVA main effects were statistically significant for musical excerpt, *F*(1, 382) = 27.171, *p* < 0.001, Pillai’s Trace = 0.066, OP = 0.999, for motion degree, *F*(4, 379) = 137.551, *p* < 0.001, Pillai’s Trace = 0.592, OP = 1, and for musical expertise, *F*(1, 382) = 3.988, *p* = 0.047, OP = 0.513, on the OPE scores for the AV condition. In addition, there was a significant interaction between musical expertise and musical excerpt, *F*(1, 382) = 12.772, *p* < 0.001, Pillai’s Trace = 0.032, OP = 0.946, and between musical excerpt and motion degree, *F*(4, 379) = 35.177, *p* < 0.001, Pillai’s Trace = 0.271, OP = 1 (Table 2).

**Table 2:** Three-way multivariate analysis of variance (MANOVA) 2 × 2 × 5 for the overall performance scores in audio–visual condition

Factor	Pillai’s Trace	*F*	*df* between	*df* error	*p*
(A) Musical expertise	0.010	3.988	1	382	0.047
(B) Musical excerpt	0.066	27.171	1	382	< 0.001
(C) Motion degree	0.592	137.551	4	379	< 0.001
A × B	0.032	12.772	1	382	< 0.001
A × C	0.014	1.338	4	379	0.255
B × C	0.271	35.177	4	379	< 0.001
A × B × C	0.017	1.596	4	379	0.175

#### Non-musicians’ ratings differ significantly in the two musical excerpts

**Fig. 1:**
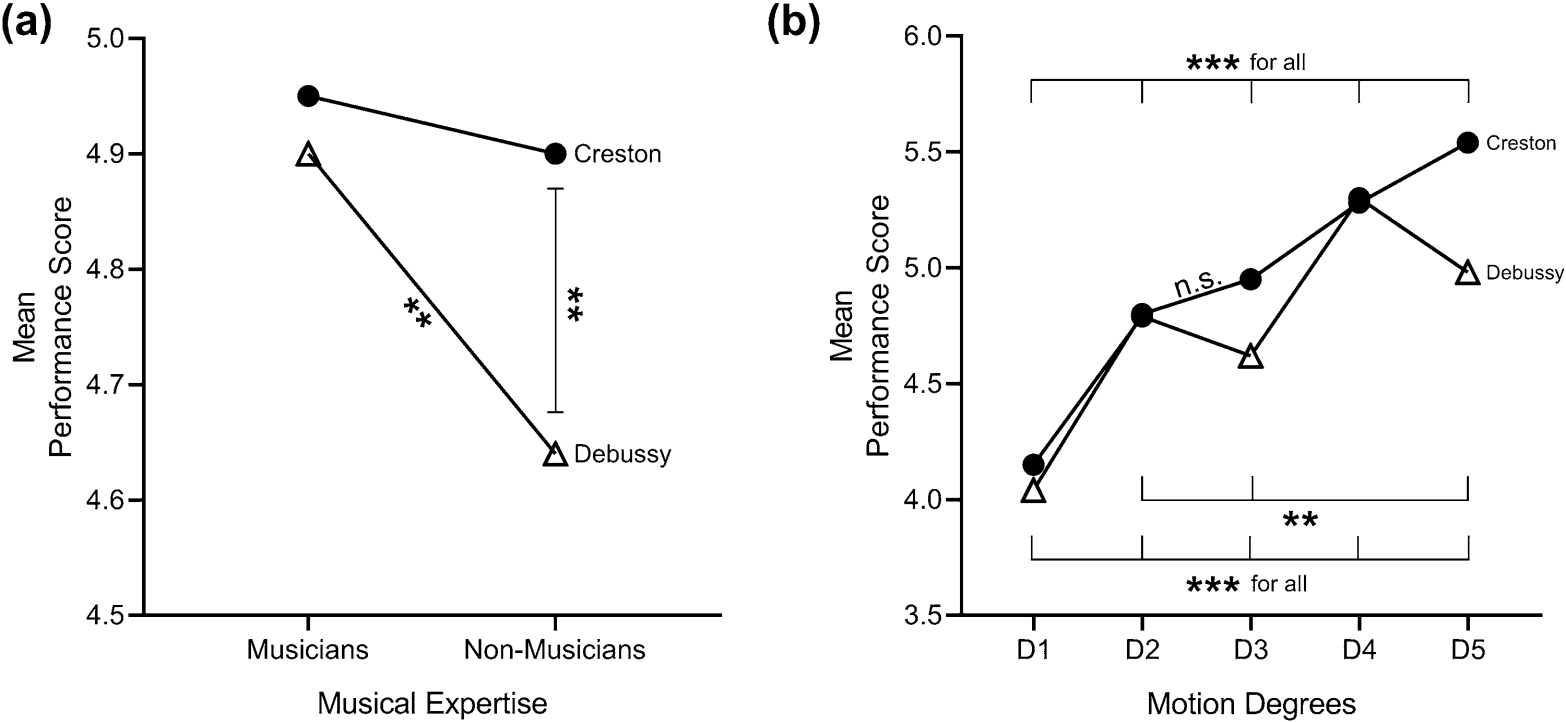
**a** Interactions for musical expertise and musical excerpt, **b** interactions for motion degree and musical excerpt (in Creston, significant differences were found for all comparisons but motion degrees 2–3; in Debussy, significant differences were found for all comparisons with *** and 2–3 and 2–5 with **). Significance levels are reported as follows: **p < 0.01, ***p < 0.001. Alpha level for all tests was set at 0.05

Subsequent tests of the interaction of musical expertise and musical excerpt showed that the non-musicians rated the Creston excerpt significantly higher than the Debussy (Δ = 0.263), *F*(1, 382) = 38.6, *p* < 0.001, Pillai’s Trace = 0.092, OP = 1. For the musicians, no significant differences were found between the ratings of the two musical excerpts, *F*(1, 382) = 1.343, *p* = 0.247, Pillai’s Trace = 0.004, OP = 0.212 (see Table 3, Fig. 1a).

**Table 3:** Means (and standard deviations) for overall performance scores, and main univariate *F* values for each musical excerpt

Musical excerpt	Musical expertise		Motion degree					
	Musician	Non-musician	D1^a^	D2^b^	D3^c^	D4^d^	D5^e^	*F*(4, 379)
Creston	4.95 (1.24)	4.9 (1.18)	4.04 (1.16)	4.8 (1.18)	4.95 (1.08)	5.28 (1.10)	5.54 (1.04)	123.098***
Debussy	4.90 (1.21)	4.64 (1.05)	4.15 (1.12)	4.79 (1.06)	4.62 (1.01)	5.3 (1.08)	4.98 (1.02)	89.454***
*F*(1,382)	1.343	38.6**						

**p < 0.01; ***p < 0.001

^a^Minimal

^b^In Creston, head nods and in Debussy, anteroposterior sway

^c^In Creston, knee and trunk flexion and in Debussy, mediolateral sway

^d^Flap

^e^Exaggerated

#### Creston’s ratings, unlike Debussy’s, increase as a function of quantity of motion

Follow-up tests of the interaction of musical excerpt and motion degree found significant differences in the motion degree ratings for the Creston excerpt, *F*(4, 379) = 123.098, *p* < 0.001, Pillai’s Trace = 0.565, OP = 1, and for the Debussy excerpt, *F*(4, 379) = 89.454, *p* < 0.001, Pillai’s Trace = 0.486, OP = 1.

This interaction was followed by Holm–Bonferroni pairwise comparisons. For the Creston, the score hierarchy was D5 > D4 > D3 > D2 > D1, with the following significant differences: D5 scored significantly higher than all others (then D1, Δ = 1.494; D2, Δ = 0.733; D3, Δ = 0.585; D5, Δ = 0.252; in all, *p* < 0.001); D4 was higher than D1, Δ = 1.242; D2, Δ = 0.481; D3, Δ = 0.333; in all, *p* < 0.001; D3 was higher than D1, Δ = 0.909, *p* < 0.001; and D2 was higher than D1, Δ = 0.761, *p* < 0.001. For the Debussy, the score hierarchy was D4 > D5 > D2 > D3 > D1, with the following significant differences: D4 (Flap) scored significantly higher than all others (than D1, Δ = 1.152; D2, Δ = 0.505; D3, Δ = 0.675; D5, Δ = 0.32; in all, *p* < 0.001); D5 was higher than D1, Δ = 0.832, *p* < 0.001; D2, Δ = 0.185,* p* = 0.004; D3, Δ = 0.355, *p* < 0.001; D2 (AP Sway) was higher than D1, Δ = 0.647, *p* < 0.001 and D3, Δ = 0.17,* p* = 0.008; and D3 was higher than D1, Δ = 0.477, *p* < 0.001. Descriptives are presented in Table 3, for a graphical representation, see Fig. 1b.

### Sensory dominance in multimodal presentation

The four-way MANOVA main effects were statistically significant for musical excerpt *F*(1, 382) = 54.916, *p* < 0.001, Pillai’s Trace = 0.126, OP = 1, for motion degree *F*(4, 379) = 221.562, *p* < 0.001, Pillai’s Trace = 0.7, OP = 1, for sensory mode, *F*(2, 381) = 212.087, *p* < 0.001, Pillai’s Trace = 0.527, OP = 1, and for musical expertise *F*(1, 382) = 6.22, *p* = 0.013, OP = 0.701, on the OPE scores for the A, AV and V conditions. Considering that the focus of this section is on differences between sensory modes, we further focus on the interactions including this factor (others can be consulted in Appendix 1 of the SI).

The two-way interaction between motion degree and sensory mode was statistically significant, *F*(8, 375) = 70.112, *p* < 0.001, Pillai’s Trace = 0.599, OP = 1. The three-way interaction between sensory mode, musical excerpt and motion degree was also statistically significant, *F*(8, 375) = 45.138, *p* < 0.001, Pillai’s Trace = 0.491, OP = 1. Finally, the four-way interaction between musical expertise, sensory mode, musical excerpt and motion degree was statistically significant, *F*(8, 375) = 2.472, *p* = 0.013, Pillai’s Trace = 0.05, OP = 0.905 (Table 4).

**Table 4:** Four-way multivariate analysis of variance (MANOVA) 2 × 2 × 5 × 3 for the overall performance scores in audio-only, audio–visual and visual-only conditions

Factor	Pillai’s Trace	*F*	*df* between	*df* error	*p*
(A) Musical expertise	0.016	6.22	1	382	0.013
(B) Musical excerpt	0.126	54.916	1	382	< 0.001
(C) Motion degree	0.700	221.562	4	379	< 0.001
(D) Sensory mode	0.527	212.087	2	381	< 0.001
A × B	0.046	18.58	1	382	< 0.001
A × C	0.067	6.825	4	379	< 0.001
A × D	0.001	0.167	2	381	0.846
B × C	0.306	41.746	4	379	< 0.001
B × D	0.003	0.595	2	381	0.552
C × D	0.599	70.112	8	375	< 0.001
A × B × C	0.026	2.55	4	379	0.039
A × B × D	0.000	0.054	2	381	0.947
A × C × D	0.034	1.652	8	375	0.109
B × C × D	0.491	45.138	8	375	< 0.001
A × B × C × D	0.050	2.472	8	375	0.013

#### Main effects: musicians give increased ratings, Creston excerpt and audio-only condition score higher

Subsequent univariate tests revealed that musicians gave significantly higher scores than non-musicians (respectively, *M* = 4.83, *M* = 4.66, Δ = 0.17), *F*(1, 382) = 6.22, *p* = 0.013, OP = 0.701, and that the Creston excerpt was significantly higher rated that the Debussy (respectively, *M* = 4.84, *M* = 4.66, Δ = 0.173), *F*(1, 382) = 54.916, *p* < 0.001, OP = 1. Regarding sensory mode, differences were found between all conditions, *F*(1, 382) = 54.916, *p* < 0.001, Pillai’s Trace = 0.527, OP = 1. A condition scored significantly higher than AV (respectively, *M* = 5.08, *M* = 4.85, Δ = 0.238, *p* < 0.001) and V (M = 4.32, Δ = 0.768, *p* < 0.001), and AV higher than V (Δ = 0.53, *p* < 0.001). Considering that motion degrees are dependent on the musical excerpt, we did not follow their isolated effect.

#### Four-way interaction: visual dominance in both groups for Creston, but musicians shift to auditory dominance in Debussy

Since the four-way interaction between all factors was significant, we focussed the analysis on these results, as they summarise each of the previous interaction stages. Follow-up tests revealed a significant effect of the motion degree for musicians and non-musicians, in each musical excerpt and each sensory mode (for all, *p* < 0.001, OP = 1, see Table 5 for descriptives and results of each test).

**Table 5:** Means (and standard deviations) for overall performance scores, and main multivariate *F* values for the effect of motion degree within each level combination of the other effects (musical expertise, sensory mode, musical excerpt)

Musical expertise	Sensory mode	Musical excerpt	Motion degree					
			1^a^	2^b^	3^c^	4^d^	5^e^	*F*(4, 379)
Musician	A	Creston	4.97 (1.07)	5.51 (1.10)	5.01 (1.15)	5.30 (1.02)	5.28 (1.05)	14.281***
		Debussy	4.73 (1.04)	5.03 (1.11)	4.91 (1.10)	5.54 (1.04)	5.36 (1.12)	30.018***
	AV	Creston	4.03 (1.18)	4.85 (1.21)	5.04 (1.08)	5.31 (1.10)	5.51 (1.06)	62.213***
		Debussy	4.24 (1.13)	4.96 (1.11)	4.72 (1.10)	5.49 (1.12)	5.09 (1.07)	52.203***
	V	Creston	2.95 (1.15)	4.13 (1.26)	4.75 (1.23)	5.13 (1.14)	5.29 (1.20)	122.806***
		Debussy	3.34 (1.23)	4.64 (1.15)	4.01 (1.16)	4.94 (1.26)	4.95 (1.17)	88.738***
Non-musician	A	Creston	5.09 (1.06)	5.29 (1.02)	4.92 (1.02)	5.11 (0.98)	5.36 (0.99)	10.345***
		Debussy	4.64 (1.02)	4.68 (0.89)	4.91 (1.02)	5.14 (0.95)	4.90 (0.98)	12.826***
	AV	Creston	4.05 (1.14)	4.76 (1.16)	4.86 (1.07)	5.26 (1.09)	5.56 (1.01)	62.513***
		Debussy	4.05 (1.10)	4.63 (0.97)	4.53 (0.90)	5.11 (1.00)	4.87 (0.96)	28.534***
	V	Creston	2.93 (1.16)	3.95 (1.20)	4.76 (1.14)	4.94 (1.11)	5.16 (0.99)	110.351***
		Debussy	3.27 (1.11)	4.15 (1.15)	3.79 (1.08)	4.44 (1.16)	4.80 (1.14)	61.417***

*A* audio-only, *AV* audio–visual, *V* visual

***p < 0.001, observed power = 1

^a^Minimal

^b^In Creston, head nods and in Debussy, anteroposterior sway

^c^In Creston, knee and trunk flexion and in Debussy, mediolateral sway

^d^Flap

^e^Exaggerated

**Fig. 2:**
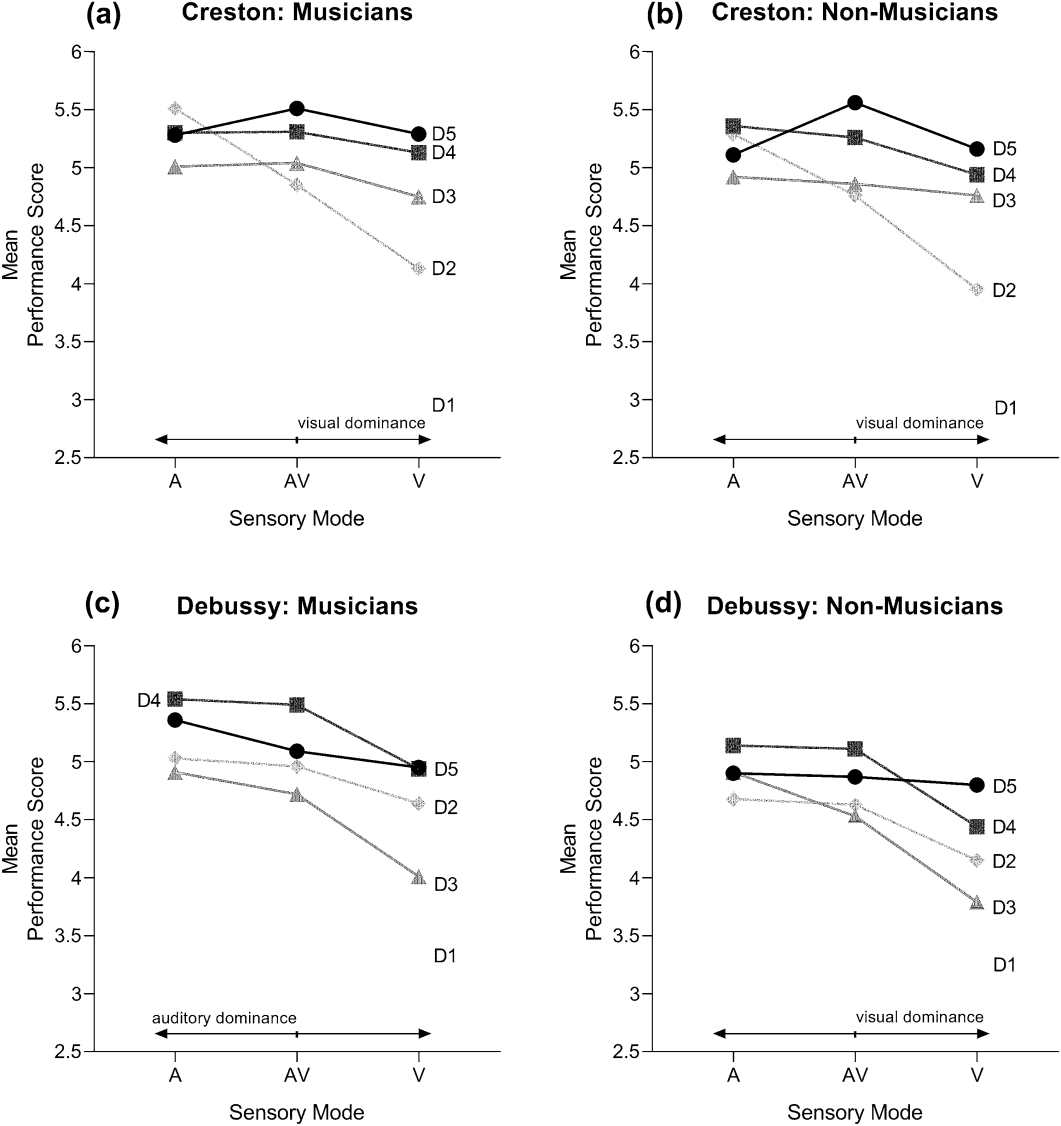
Interaction plots for sensory mode (*A* audio-only, *AV* audio–visual, *V* visual) and motion degrees in each musical excerpt and group of musical expertise

**Fig. 3:**
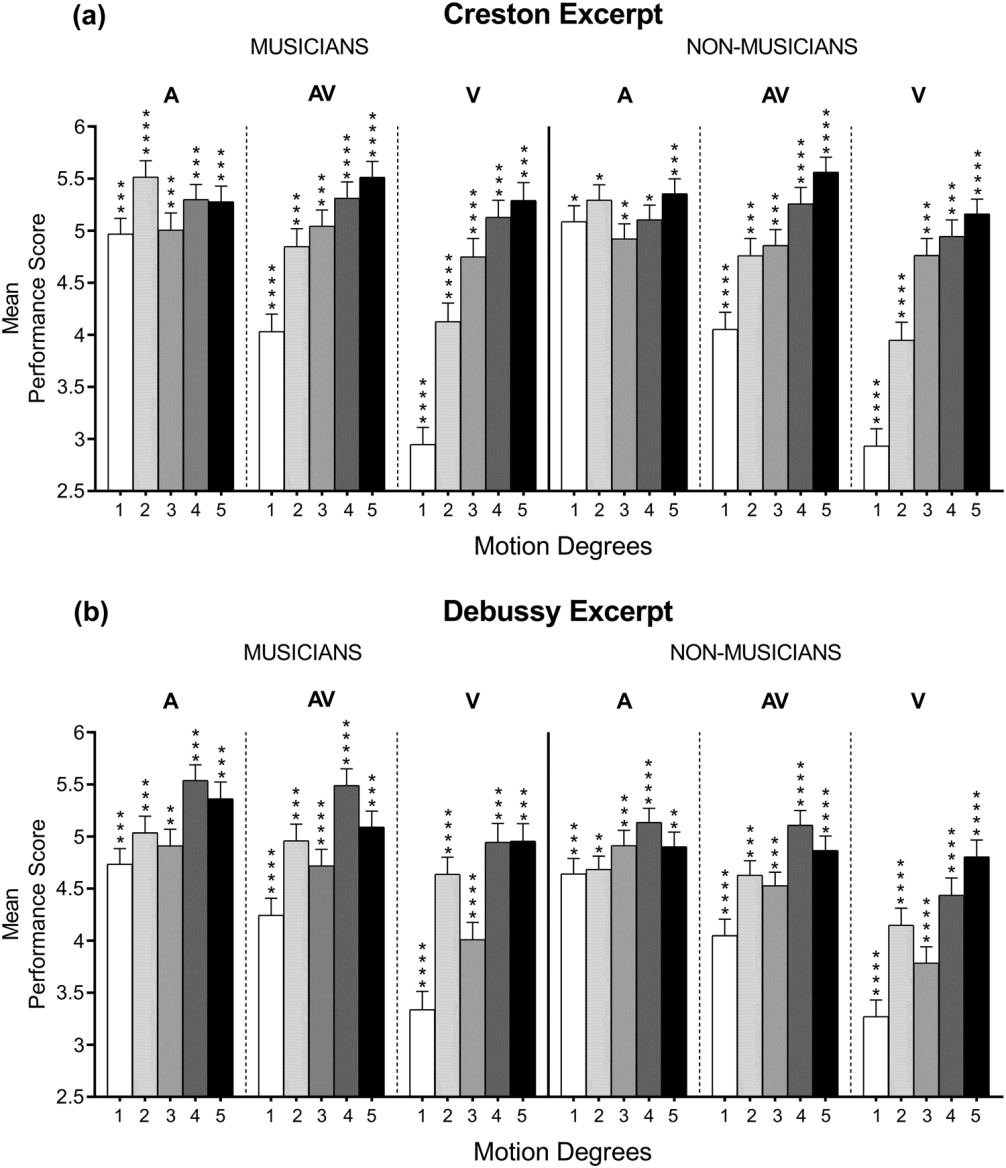
Mean performance scores (error bars represent 95% confidence intervals) for each motion degree (1…5), in each sensory mode and musical expertise group. Asterisks (*) represent the number of significantly different comparisons found between that motion degree and others within the condition (for example, in a), Musicians A, bar 1 contains ***, meaning it was significantly different than three other motion degrees within that sensory mode)

We conducted Holm–Bonferroni pairwise comparisons to examine the differences between motion degrees in each combination level. As reported in detail in Table 6, in which we cite mean differences and significance levels, most comparisons were statistically significant. In the Creston excerpt, for the musician group, A condition, the score hierarchy was D2 > D5 > D4 > D3 > D1. However, in the AV and V condition, it changed to D5 > D4 > D3 > D2 > D1. Similarly for the non-musician group, in the Creston excerpt, A condition, the score hierarchy was D5 > D2 > D4 > D1 > D3, differing in AV and V condition to D5 > D4 > D3 > D2 > D1. In the Debussy excerpt, for the musician group, in A and AV conditions, the score hierarchy was D4 > D5 > D2 > D3 > D1. In the V condition, however, it changed to D5 > D4 > D2 > D3 > D1. Diverging patterns were found for the non-musician group, in the same excerpt: in the A condition, the score hierarchy was D4 > D3 > D5 > D2 > D1, whereas in the AV condition, it was D4 > D5 > D2 > D3 > D1, and in the V condition, D5 > D4 > D2 > D3 > D1. Figures 2 and 3 present graphical representations of these findings.

**Table 6:** Post hoc multiple comparisons using Holm–Bonferroni correction. Mean differences shown

Creston excerpt									
Musician, audio-only condition					Non-musician, audio-only condition				
	D1	D2	D3	D4		D1	D2	D3	D4
D2	0.547***				D2	0.217			
D3	0.038	− 0.509***			D3	− 0.167	− 0.373***		
D4	0.311**	− 0.236*	0.273**		D4	0.019	− 0.188	0.186	
D5	0.33***	− 0.217*	0.292**	0.019	D5	0.269**	0.062	0.436***	0.25**

Musician, audio–visual condition					Non-musician, audio–visual condition				
	D1	D2	D3	D4		D1	D2	D3	D4
D2	0.816***				D2	0.707***			
D3	1.014***	0.198			D3	0.804***	0.097		
D4	1.28***	0.464***	0.266*		D4	1.205***	0.498***	0.401***	
D5	1.481***	0.665***	0.467***	0.201*	D5	1.507***	0.8***	0.703***	0.302***

Musician, visual-only condition					Non-musician, visual-only condition				
	D1	D2	D3	D4		D1	D2	D3	D4
D2	1.179***				D2	1.016***			
D3	1.802***	0.623***			D3	1.828***	0.813***		
D4	2.181***	1.002***	0.378***		D4	2.01***	0.995***	0.182	
D5	2.342***	1.663***	0.54***	0.161	D5	2.227***	1.212***	0.399***	0.217*

Debussy excerpt									
Musician, audio-only condition					Non-musician, audio-only condition				
	D1	D2	D3	D4		D1	D2	D3	D4
D2	0.3***				D2	0.042			
D3	0.177	− 0.123			D3	0.271***	0.229*		
D4	0.804***	0.503***	0.675***		D4	0.493***	0.451***	0.222*	
D5	0.627***	0.326***	0.455***	− 0.177	D5	0.26*	0.219	− 0.01	− 0.233*

Musician, audio–visual condition					Non-musician, audio–visual condition				
	D1	D2	D3	D4		D1	D2	D3	D4
D2	0.714***				D2	0.58***			
D3	0.474***	− 0.24**			D3	0.479***	− 0.101		
D4	1.245***	0.531***	0.771***		D4	1.059***	0.479***	0.58***	
D5	0.845***	0.132	0.372***	− 0.399***	D5	0.818***	0.238*	0.58***	− 0.241*

Musician, visual-only condition					Non-musician, visual-only condition				
	D1	D2	D3	D4		D1	D2	D3	D4
D2	1.3***				D2	0.875***			
D3	0.674***	− 0.627***			D3	0.514***	− 0.361***		
D4	1.608***	0.307**	0.934***		D4	1.163***	0.288**	0.649***	
D5	1.618***	0.318**	0.944***	0.01	D5	1.531***	0.656***	1.017***	0.368***

Significance levels: * p < 0.05; ** p < 0.01; *** p < 0.001

Significance levels: * *p* < 0.05; ** *p* < 0.01; *** *p* < 0.001.

## Discussion

In this study, we investigated the role of musicians’ body behaviour in the observers’ perception of music performance quality. It is well established that body movements influence how the audience evaluates (e.g. Broughton & Stevens, 2009), feels (e.g. Coutinho & Scherer, 2017) and even hears (e.g. Schutz & Lipscomb, 2007) performances. We built on previous research by increasing the number of motion degrees included in our design, a suggestion that has been placed before (Silveira, 2014), which allowed us to further inspect how quantity and quality of motion take part in performance evaluation. In addition, we used stimuli presenting two contrasting musical excerpts to understand if the motion degrees perceived as optimal vary depending on musical style. Finally, we compared ratings in audio-only (A), audio–visual (AV) and visual-only (V) conditions to test for sensory dominance. To analyse the potential effects of musical expertise, both musician and non-musician participants took part in the experiment.

Our first set of hypotheses (H1…H3) focussed on body behaviour in AV perception, the most proximal condition to real performance settings. We found that musical style has a determinant effect on whether observers’ build their evaluation based on the quantity or quality of motion. For the positive-valenced Creston excerpt (fast, energetic, major mode), the ratings went up as the quantity of motion increased (D1 < D2 < D3 < D4 < D5), whereas for the negative-valenced Debussy excerpt (slow, melancholic, harmonically complex), the quality of motion was more important than the quantity (D1 < D3 < D2 < D5 < D4). These results suggest that positive-valenced music is perceived as matching high motion profiles, and negative-valenced music yields a different music-movement match perception, in which specific gestural behaviours can be perceived as more adequate. In this sense, our findings partially align with other studies in which better performative ratings were recorded in conditions with increased amounts of movement (Broughton & Stevens, 2009; Bugaj et al., 2019; Burger & Wöllner, 2023; Davidson, 1993; Grady & Gilliam, 2020; Juchniewicz, 2008; Moura et al., 2023; Nápoles et al., 2022; Nusseck & Wanderley, 2009; Silveira, 2014; Trevor & Huron, 2018; Van Zijl & Luck, 2013). However, we expand on these studies by demonstrating that such observation is only applicable to the case of happy, energetic, and harmonically familiar music. Concomitantly, in the study by Trevor and Huron (2018), when asked to adjust the movements of musicians’ stick figures to create the best performances, participants favoured augmented movement for fast technical passages and only slightly above normal movement for slower lyrical passages. Yet, here, in the Debussy, the flap motion degree (D4) was perceived as the most adequate, and it presented the second-highest QoM value. Also, anterior–posterior sway (D2) scored higher than mediolateral sway (D3), although it held lower QoM. Based on these results, we infer that, for the case of negative-valenced music, isolated motion types are perceived as more adequate than overall exaggerated motion, possibly due to the way they are executed in association with the music (i.e. based on visual inspection, the performer’s flaps reflected phrasing intentions). Further research is needed to better understand this effect. Whatsoever, common to both excerpts, the minimal movement degree (D1) was the lowest scored, suggesting that, despite the musical style, performances with restricted motion are perceived as less expressive, professional and overall worse. This result directly aligns with previous studies in which low movement conditions were the lowest rated (Davidson, 1993; Moura et al., 2023; Weiss et al., 2018). Hence, if listening to moving musical forms whilst watching static body motion is perceived as poorer music performance, we conclude, after previous work (Id et al., 2019; Thompson et al., 2005), that a certain level of congruency between sound and visuals is required to enhance perceptual experiences.

Musical expertise did not have a significant effect on the AV motion degree ratings, suggesting that musicians and non-musicians share proximal conceptions regarding the match between music and body behaviour. Nevertheless, it had a significant effect on the ratings of the musical excerpts. Whilst non-musicians gave significantly higher ratings to Creston in comparison to Debussy, musicians gave proximal ratings to both, indicating that they can equally appreciate performances of analogous quality levels (here, expert performances) independently of their personal musical preference. Studies have demonstrated that musical training translates into a greater preference towards musical complexity (Matthews et al., 2019; North & Hargreaves, 1995; Witek et al., 2023). Furthermore, experts develop distancing mechanisms that attenuate preliminary emotional reactions in art appreciation (Leder & Schwarz, 2017; Leder et al., 2014), allowing them to focus on aesthetic qualities related to stylistic and formal aspects (Scherer, 2005). This phenomenon enables experts, for example, to appreciate negatively valenced art more than non-experts (Leder et al., 2014), as they are able to detach from the emotional impact of the artwork. These findings support why musicians gave similar ratings across excerpts, even though the Debussy presented a more complex harmonic and rhythmical structure. On the other hand, in Western classical music, research has shown that listeners typically associate fast tempo and major modes with happiness, whereas sad-perceived music is usually slow and minor (Schellenberg et al., 2008). Up-tempo, major excerpts are higher rated (Burger & Wöllner, 2023; Husain et al., 2002; Schellenberg et al., 2008; Webster & Weir, 2005) and provoke greater levels of post-listening enjoyment (Thompson et al., 2001). In this sense, non-musicians followed a natural tendency to prefer joyous, energetic music rather than the complex Debussian excerpt, whose compositional style departs from traditional diatonicity and can ultimately sound unfamiliar to unexperienced listeners (Laneve et al., 2023).

To investigate our second set of hypotheses (H4, H5), we analysed the differences between motion degrees in A, AV and V conditions. The main finding deriving from the interaction analysis was that, whereas both musicians and non-musicians displayed patterns of visual dominance for the Creston excerpt, for the Debussy, musicians shifted to auditory dominance. Again, the musical style had a significant effect on multisensory perception. In the Creston, our results align with studies demonstrating that expert musicians make equally strong use of vision as novices (Tsay, 2013, 2014). Furthermore, the pattern of visual dominance found in both groups aligns with several studies showing that visual cues exceed auditory ones in music performance evaluation (Broughton & Stevens, 2009; Coutinho & Scherer, 2017; Lange et al., 2022; Pope, 2019; Schutz & Lipscomb, 2007; Wapnick et al., 2004). In the Debussy, inversely, we observed that non-musicians relied more on the visuals, as suggested by other group of studies (Davidson, 1995; Davidson & Correia, 2002; Huang & Krumhansl, 2011). Repertoire following harmonically innovative musical systems, such as the Debussy, can potentially induce more ambiguous listening experiences (Laneve et al., 2023). In this sense, we hypothesise that, due to the superior compositional and emotional complexity of the Debussy, musicians were absorbed by the auditory information, focussing their attention on the sound. In the study by Kawase and Obata (2016), observers’ visual attention was directed to the main melodic parts, hence shaped by sound. Accordingly, sonic information has revealed to be prevalent in emotion-related perceptual tasks (Shoda & Adachi, 2016; Vines et al., 2011), specifically in identifying music with negative valence (Van Zijl & Luck, 2013). Our results also reinforce the idea that visual and auditory cues interact in complex ways depending on the context (Coutinho & Scherer, 2017; Li et al., 2021; Vuoskoski et al., 2014), highlighting the need to replicate such findings with an expanded repertoire. For example, in Li and Colleagues (2021), visual condition was effective in communicating tense and relaxed timbres, but other timbres were heavily dependent on the performer. Accordingly, we believe that sensory dominance dynamically transfers depending on the musical style.

The influence of body movement in evaluation is further validated, in some cases, through oscillations in the hierarchies of motion degrees between sensory conditions. For example, in the Creston excerpt, D2 was rated first (musicians) and second (non-musicians) in A condition, drastically falling to fourth in AV (both groups). Furthermore, in the AV condition, the ratings of both groups increased as a function of the QoM. This reinforces that performances of positively valenced music are prejudiced when combined with constrained motion profiles and, hence, augmented when combined with exaggerated amounts of motion. However, in contrast to what we had initially predicted, the A condition received higher overall ratings than the AV and V, respectively. Considering that the AV condition provided multimodal input, it would be expected that it would be more appealing, considering previous views on the visual dimension as a driving force for audiences to attend live performances rather than consume recordings (Bergeron & Lopes, 2009; Cook, 2008; Platz & Kopiez, 2012). Yet, live performances also involve a dimension of human interaction which is not possible to account for in studies of this kind. The decrease in AV and V scores can possibly be related to the use of an avatar to represent human performers, which can translate into a less natural way of watching performances. Although it is true that some of the studies we refer to used regular video (e.g. Lange et al., 2022; Nápoles et al., 2022; Silveira, 2014; Tsay, 2013, 2014), it is well known that it does not allow for control of confounders such as physical appearance or dress style (e.g. Griffiths, 2008; Urbaniak & Mitchell, 2022). Hence, we followed the methodology of using kinematic, de-characterised displays (e.g. Davidson, 1993; Vinken & Heinen, 2022; Weiss et al., 2018), to homogenise the set of stimuli. In the future, experiments using humanoid avatars or even *in loco* experiments involving live performances (e.g. Coutinho & Scherer, 2017) would be desirable to pursue this result.

One limitation of our study relates to the fact that the musical excerpts used, retrieved from emblematic saxophone works, combine multiple components, including tempo and rhythm, tonality, or motific structure. Based on the associations between musical aspects and valence reported in previous research (Husain et al., 2002; Scherer, 2005; Thompson et al., 2001; Webster & Weir, 2005), we treated musical style as a global concept encompassing these various features. Consequently, it was not possible to assert if the effect of the music was due to the interaction of factors together or if certain factors had stronger isolated contributions. For example, Burnham and Colleagues (2021) found that pitch direction mediated the ability of participants to identify major and minor modes, ultimately associated with positive and negative constructs. In this sense, it would be interesting to conduct follow-up research under a manipulation paradigm, thus controlling the stimuli, for instance, by presenting them at gradual levels of tempi or transposing the excerpts to other modes. Nevertheless, as previously stated (Battcock & Schutz, 2019, 2022), we highlight the importance of using renown repertoire in perceptual studies. First, it allows for a better understanding of the real, everyday concert experience, involving the exposition to complex music with its multiple layers acting cumulatively as planned by its composer. Second, using original repertoire allows for the preservation of the natural expressive behaviour of the performers and the creation of knowledge that is directly applicable to their instrumental practise.

On this basis, we conclude by emphasising that body behaviour has a strong impact on musical performance communication, particularly considering its interaction with musical style. Positively and negatively valenced musical excerpts result in distinct conceptions of optimal motion amongst observers. Furthermore, we validate that non-musician prioritise visuals over sound independently from musical context, whereas musicians turn to sound in contexts with increased complexity. We strongly motivate further research, including other musical excerpts, genres and instruments, to better define the optimal motion degrees according to music categories. The applications of our findings can be transposed into music performance pedagogy, allowing performers to adapt their motion style to the repertoire being performed. The resulting knowledge can also contribute to the development of applications for performance analysis and real-time motion monitoring for musicians and music students. More broadly, these findings provide insights towards the conception of concert models integrating multimodality as a means for enhancing listening experiences and promoting musical understanding, engagement, and emotionality.

### Supplementary Information

Below is the link to the electronic supplementary material.

Supplementary file1 (DOCX 96 KB)

Supplementary file2 (MOV 2137 KB)

Supplementary file3 (MOV 1767 KB)

Supplementary file4 (MOV 2468 KB)

Supplementary file5 (MOV 1854 KB)

## Data Availability

Examples of the stimuli used in this study are openly available as Supporting Information. The dataset generated and analysed in this study is available from the corresponding author upon reasonable request.
