# Supplementary material for: Increased body movement equals better performance? Not always! Musical style determines motion degree perceived as optimal in music performance
Source: Psychol Res. 2024 Feb 8;88(4):1314–30. doi: 10.1007/s00426-024-01928-x (PMC11142955; doi:10.1007/s00426-024-01928-x)
Supplement: Supplementary file 1 — Supplementary file1 (DOCX 96 KB) [file 426_2024_1928_MOESM1_ESM.docx]

# **Supplementary Information**

# Increased body movement equals better performance? Not always! Musical style determines motion degree perceived as optimal in music performance

Nádia Moura^1^* (ORCID: 0000-0002-4618-4146), Pedro Fonseca^2^ (ORCID: 0000-0002-4885-4924), João Paulo Vilas-Boas^2,3^ (ORCID: [0000-0002-4109-2939](https://orcid.org/0000-0002-4109-2939)), Sofia Serra^14^ (ORCID: 0000-0001-7052-860X)

^1^School of Arts, Research Centre in Science and Technology of the Arts, Universidade Católica Portuguesa, Porto, Portugal

^2^Porto Biomechanics Laboratory, Faculty of Sport, University of Porto, Porto, Portugal

^3^Centre of Research, Education, Innovation and Intervention in Sport, Faculty of Sport, University of Porto, Porto, Portugal

^4^Instituto de Etnomusicologia – Centro de Estudos em Música e Dança, [Departamento de Comunicação e Arte, Universidade de Aveiro,](http://www.inetmd.pt/index.php/inet-md/polos/deca) Aveiro, Portugal

*Corresponding author: nmoura@ucp.pt


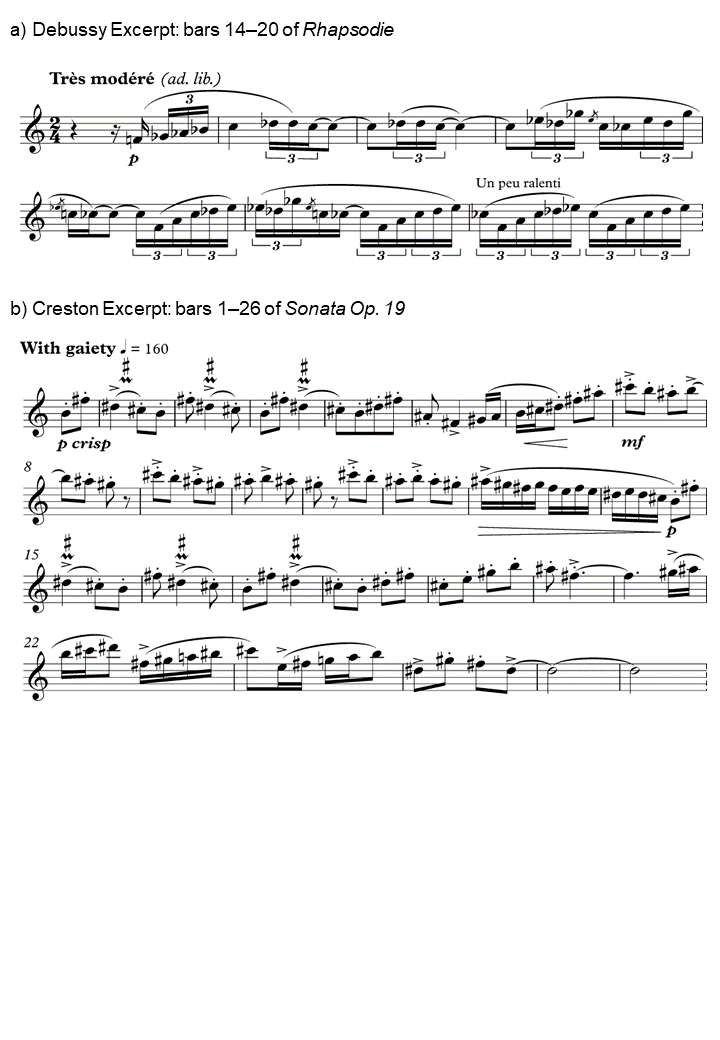


**Fig. 1** Musical scores of the two excerpts used in this study: a) Debussy Excerpt (Debussy, 1998) and b) Creston Excerpt (Creston, 1945).

References:

Creston, P. (1945). *Sonata Opus 19 for Eb Alto Saxophone*. Templeton Publishing.

Debussy, C. (1998). *Rhapsodie pour orchestre et saxophone*. Henry Lemoine.

**Table 1** Quantity of Motion (QoM) values (mean and standard deviation) per body part, in each stimulus used in this study.

|  |  | **Quantity of Motion (QoM) in mm/s** | | | | | | | | |
| --- | --- | --- | --- | --- | --- | --- | --- | --- | --- | --- |
| Musical  Excerpt | Stimuli | Head | Torso | Right Elbow | Left Elbow | Right Knee | Left Knee | Right Foot | Left Foot | Sax Bell |
| Debussy | D1  Minimal | 49.62 ± 25.54 | 52.82 ±  23.74 | 68.22 ±  38.56 | 66.29 ±  26.38 | 30.11 ± 15.89 | 32.64 ±  18.36 | 3.15 ±  3.18 | 2.66 ±  2.32 | 62.65 ±  32.47 |
|  | D2  AP Sway | 98.87 ±  59.16 | 86.8 ±  48.76 | 96.50 ±  55.57 | 120.65 ±  62.42 | 50.92 ±  40.34 | 36.3 ±  23.52 | 18.1 ±  23.05 | 5.92 ±  5.97 | 114.84 ± 61.83 |
|  | D3  ML Sway | 130.93 ± 59.45 | 116.51 ± 57.92 | 105.44 ± 56.74 | 148.66 ±  71.26 | 52.47 ± 28.7 | 54.64 ± 28.26 | 12.65 ± 11.51 | 10.78 ± 12.85 | 159.24 ± 90.27 |
|  | D4  Flap | 155.25 ± 104 | 112.28 ± 83.42 | 115.47 ± 78.22 | 193.60 ± 124.15 | 50.26 ± 40.67 | 54.38±  50.67 | 9.28 ±  12.86 | 10 ±  19.66 | 175.71 ± 121.48 |
|  | D5  Exaggerated | 155.17 ± 93.06 | 123.58 ±  78.3 | 135.08 ±  83.57 | 141.89 ±  73.58 | 69.23 ±  48.01 | 60.12 ±  41.03 | 20.80 ±  48.18 | 12.98 ±  54.21 | 195.72 ± 103.2 |
| Creston | D1  Minimal | 34.92±  21.66 | 30.25 ±  18.12 | 42.97 ±  27.83 | 40.01 ±  22.9 | 19.53 ±  16.04 | 17.56 ±  16.73 | 1.62 ±  1.36 | 3.57 ±  3.86 | 33.27 ±  20.61 |
|  | D2  Head Nod | 137.05 ± 96.08 | 96.32 ±  81.99 | 108.86 ±  100.27 | 114.2 ±  94.96 | 69.88 ±  56.66 | 69.74 ±  49.88 | 21.34 ± 43.2 | 10.67 ± 25.54 | 95.44 ±  96.63 |
|  | D3  Trunk + Knee Flexion | 239.14 ±  145.49 | 175 ±  103.9 | 162.09 ±  100.51 | 175.51 ±  98.1 | 92.32 ±  72.13 | 90.78 ±  70.01 | 17.72 ±  65.68 | 27.02 ±  48.88 | 223.05 ±  150.15 |
|  | D4  Flap | 233.63 ±  144.07 | 163.64 ±  96.16 | 170.46 ± 95.89 | 331.48±  201.14 | 89.44 ±  65.27 | 99.83 ±  60.11 | 8.49 ±  36.73 | 36.24 ±  47.86 | 202.74 ±  144.24 |
|  | D5  Exaggerated | 379.12 ±  194.39 | 245.63 ±  124.12 | 248.29 ±  139.56 | 323.25 ±  156.67 | 126.45 ±  76.69 | 142.21±  87.30 | 23.11 ±  21.73 | 36.57 ±  45.46 | 311.77 ±  183.65 |

**Table 2** Pearson correlation coefficients between the rating scores of Expressiveness (EXP), Quality (QUAL) and Professionalism (PRO) for the stimuli used in this study (Stimulus 1…10, A: Audio-only, AV: Audio-visual, V: Visual-only).

|  | **Musicians** | | | **Non-Musicians** | | |
| --- | --- | --- | --- | --- | --- | --- |
| Stimuli | EXP*PRO | EXP*QUAL | PRO*QUAL | EXP*PRO | EXP*QUAL | PRO*QUAL |
| A1 | 0.78** | 0.85** | 0.79** | 0.57** | 0.7** | 0.72** |
| A2 | 0.73** | 0.79** | 0.84** | 0.53** | 0.56** | 0.63** |
| A3 | 0.79** | 0.78** | 0.84** | 0.61** | 0.71** | 0.69** |
| A4 | 0.74** | 0.77** | 0.82** | 0.57** | 0.58** | 0.68** |
| A5 | 0.77** | 0.78** | 0.82** | 0.55** | 0.7** | 0.74** |
| A6 | 0.69** | 0.72** | 0.85** | 0.59** | 0.71** | 0.75** |
| A7 | 0.58** | 0.73** | 0.78** | 0.58** | 0.68** | 0.65** |
| A8 | 0.73** | 0.79** | 0.84** | 0.62** | 0.69** | 0.75** |
| A9 | 0.72** | 0.78** | 0.81** | 0.61** | 0.72** | 0.71** |
| A10 | 0.58** | 0.72** | 0.66** | 0.59** | 0.63** | 0.77** |
| AV1 | 0.7** | 0.84** | 0.76** | 0.61** | 0.69** | 0.68** |
| AV2 | 0.69** | 0.83** | 0.79** | 0.56** | 0.75** | 0.66** |
| AV3 | 0.73** | 0.79** | 0.83** | 0.49** | 0.58** | 0.67** |
| AV4 | 0.76** | 0.84** | 0.78** | 0.63** | 0.79** | 0.74** |
| AV5 | 0.72** | 0.76** | 0.74** | 0.56** | 0.64** | 0.7** |
| AV6 | 0.67** | 0.74** | 0.78** | 0.57** | 0.65** | 0.68** |
| AV7 | 0.69** | 0.79** | 0.79** | 0.68** | 0.76** | 0.75** |
| AV8 | 0.65** | 0.78** | 0.74** | 0.74** | 0.78** | 0.74** |
| AV9 | 0.69** | 0.81** | 0.82** | 0.7** | 0.76** | 0.74** |
| AV10 | 0.72** | 0.79** | 0.83** | 0.67** | 0.72** | 0.73** |
| V1 | 0.76** | 0.86** | 0.75** | 0.67** | 0.81** | 0.71** |
| V2 | 0.79** | 0.84** | 0.81** | 0.71** | 0.82** | 0.71** |
| V3 | 0.79** | 0.83** | 0.74** | 0.64** | 0.8** | 0.69** |
| V4 | 0.81** | 0.87** | 0.81** | 0.73** | 0.81** | 0.74** |
| V5 | 0.77** | 0.8** | 0.75** | 0.76** | 0.81** | 0.76** |
| V6 | 0.66** | 0.86** | 0.66** | 0.67** | 0.85** | 0.72** |
| V7 | 0.78** | 0.81** | 0.81** | 0.77** | 0.7** | 0.67** |
| V8 | 0.73** | 0.79** | 0.77** | 0.66** | 0.72** | 0.67** |
| V9 | 0.72** | 0.75** | 0.78** | 0.69** | 0.71** | 0.7** |
| V10 | 0.74** | 0.77** | 0.79** | 0.67** | 0.64** | 0.67** |

Significance levels are reported as follows: ** *p* < 0.001.

**Appendix 1:**

**Four-way multivariate analysis of variance (MANOVA) 2 × 2 × 5 × 3**

**Analysis of Additional Interactions**

1. *2-way interactions*
   1. Musical Expertise × Musical Excerpt

The interaction was statistically significant, F(1, 382) = 18.58, p < 0.001, Pillai’s Trace = 0.046, OP = 0.99. Subsequent tests revealed differences for the Debussy excerpt, in which musicians gave significantly higher scores than non-musicians (Δ = 0.271), F(1, 382) = 13.927, p < 0.001, η_p_^2^ = 0.035, OP = 0.961.

- 1. Musical Expertise × Motion Degree

The interaction was statistically significant, F(4, 379) = 6.825, p < 0.001, Pillai’s Trace = 0.067, OP = 0.994. However, this test grouped motion degrees with no regard to the musical excerpt or sensory mode in which they were presented, thus, it had no relevance for this study.

- 1. Musical Expertise × Sensory Mode

The interaction was not statistically significant, F(2, 381) = 0.167, p = 0.846, Pillai’s Trace = 0.306, OP = 0.076.

- 1. Musical Excerpt × Motion Degree

The interaction was statistically significant, F(4, 379) = 41.746, p < 0.001, Pillai’s Trace = 0.306, OP = 1. However, this test grouped motion degrees with no regard to the sensory mode in which they were presented, thus, it had no relevance for this study.

- 1. Musical Excerpt × Sensory Mode

The interaction was not statistically significant, F(2, 381) = 0.595, p = 0.552, Pillai’s Trace = 0.003, OP = 0.149.

- 1. Motion Degree × Sensory Mode

The interaction was statistically significant, F(8, 375) = 70.112, p < 0.001, Pillai’s Trace = 0.599, OP = 1. However, this test grouped motion degrees with no regard to the musical excerpt (i.e., Creston D3 and Debussy D3 entered together, although they represent different prominent gestures), thus, it had no relevance for this study.

1. *3-way interactions*
   1. Musical Expertise × Musical Excerpt × Motion Degree

The interaction was statistically significant, F(4, 379) = 2.55, p = 0.039, Pillai’s Trace = 0.026, OP = 0.72. Subsequent tests revealed differences for the Debussy excerpt, in which musicians gave significantly higher scores than non-musicians for D2 (Δ = 0.39), F(1, 382) = 21.918, p < 0.001, η_p_^2^ = 0.054, OP = 0.997; D4 (Δ = 0.431), F(1, 382) = 24.002, p < 0.001, η_p_^2^ = 0.059, OP = 0.998 and D5 (Δ = 0.278), F(1, 382) = 10.19, p = 0.002, η_p_^2^ = 0.026, OP = 0.89. Nevertheless, these results are of questionable relevance, as they aggregate each motion degree in the 3 sensory modes.

- 1. Musical Expertise × Musical Excerpt × Sensory Mode

The interaction was not statistically significant, F(2, 381) = 0.054, p = 0.947, Pillai’s Trace = 0.000, OP = 0.058.

- 1. Musical Expertise × Motion Degree × Sensory Mode

The interaction was not statistically significant, F(8, 375) = 1.652, p = 0.109, Pillai’s Trace = 0.034, OP = 0.725.

- 1. Musical Excerpt × Motion Degree × Sensory Mode

The interaction was statistically significant, F(8, 375) = 45.138, p < 0.001, Pillai’s Trace = 0.491, OP = 1. Follow-up tests revealed significant differences between motion degrees for all cells of the design: A condition, Creston excerpt, F(4, 379) = 21.069, p < 0.001, Pillai’s Trace = 0.182, OP = 1; A condition, Debussy excerpt, F(4, 379) = 36.865, p < 0.001, Pillai’s Trace = 0.28, OP = 1; AV condition, Creston excerpt, F(4, 379) = 123.098, p < 0.001, Pillai’s Trace = 0.565, OP = 1; AV condition, Debussy excerpt, F(4, 379) = 89.454, p < 0.001, Pillai’s Trace = 0.486, OP = 1; V condition, Creston excerpt, F(4, 379) = 231.978, p < 0.001, Pillai’s Trace = 0.71, OP = 1; V condition, Debussy excerpt, F(4, 379) = 144.432, p < 0.001, Pillai’s Trace = 0.604, OP = 1.

Subsequent multiple comparisons with Holm-Bonferroni correction were conducted. For the Creston excerpt, A condition, the score hierarchy was D2>D5>D4>D3>D1: D2 scored significantly higher than 3 others (D1, Δ = 0.377; D3, Δ = 0.441; D4, Δ = 0.212; in all *p* < 0.001); followed by D5 (higher than D1, Δ = 0.299; D3, Δ = 0.364; in both *p* < 0.001), and D4 (higher than D1, Δ = 0.165, *p* = 0.04; D3, Δ = 0.229, *p* < 0.001). In the AV condition, the hierarchy was D5>D4>D3>D2>D1: D5 scored significantly higher than all others (D1, Δ = 1.494; D2, Δ = 0.733; D3, Δ = 0.585; D4, Δ = 0.252; in all *p* < 0.001); followed by D4 (higher than D1, Δ = 1.242; D2, Δ = 0.481; D3, Δ = 0.333; in all *p* < 0.001), D3 (higher than D1, Δ = 0.909, *p* < 0.001) and D2 (higher than D1, Δ = 0.761, *p* < 0.001). In the V condition, the hierarchy was equal to AV (D5>D4>D3>D2>D1): D5 scored significantly higher than all others (D1, Δ = 2.285, D2, Δ = 1.187, D3, Δ = 0.475; in all *p* < 0.001; D4, Δ = 0.189, *p* = 0.003); followed by D4 (higher than D1, Δ = 2.095; D2, Δ = 0.998; D3, Δ = 0.28; in all *p* < 0.001), D3 (higher than D1, Δ = 1.815; D2, Δ = 0.718; in both *p* < 0.001) and D2 (higher than D1, Δ = 1.097, *p* < 0.001).

For the Debussy excerpt, A condition, the score hierarchy was D4>D5>D3>D2>D1, under the following significant differences: D4 scored significantly higher than all others (D1, Δ = 0.648; D2, Δ = 0.477; D3, Δ = 0.424; D5, Δ = 0.205; in all *p* < 0.001), followed by D5 (higher than D1, Δ = 0.444; D2, Δ = 0.273; in both *p <* 0.001; D3, Δ = 0.22, *p* = 0.001), D3 (higher than D1, Δ = 0.224, *p* < 0.001) and D2 (higher than D1, Δ = 0.171, *p* = 0.015). In the AV condition, the hierarchy was D4>D5>D2>D3>D1: D4 scored significantly higher than all others (than D1, Δ = 1.152; D2, Δ = 0.505; D3, Δ = 0.675; D5, Δ = 0.32; in all *p* < 0.001), followed by D5 (higher than D1, Δ = 0.832; D3, Δ = 0.355; in both *p* < 0.001; D2, Δ = 0.185, *p* = 0.004), D2 (higher than D1, Δ = 0.647, *p* < 0.001; D3, Δ = 0.17, *p* = 0.008), and D3 (higher than D1, Δ = 0.477, *p* < 0.001). Finally, in the V condition, the hierarchy was D5>D4>D2>D3>D1: D5 scored significantly higher than all others (D1, Δ = 1.575; D2, Δ = 0.487; D3, Δ = 0.981; in all *p* < 0.001; D4, Δ = 0.189, *p* < 0.013), followed by D4 (higher than D1, Δ = 1.385; D2, Δ = 0.298; D3, Δ = 0.792; in all *p* < 0.001), D2 (higher than D1, Δ = 1.088; D3, Δ = 0.494; in both *p* < 0.001) and D3 (higher than D1, Δ = 0.594, *p* < 0.001).

Again, these results ought to be interpreted carefully, given that the following 4-way interaction reported in the paper revealed differences between the musician and non-musician groups.
